# Sharks are the preferred scraping surface for large pelagic fishes: Possible implications for parasite removal and fitness in a changing ocean

**DOI:** 10.1371/journal.pone.0275458

**Published:** 2022-10-19

**Authors:** Christopher D. H. Thompson, Jessica J. Meeuwig

**Affiliations:** 1 Marine Futures Lab, School of Biological Sciences, University of Western Australia, Crawley, Western Australia, Australia; 2 Pristine Seas, National Geographic Society, Washington, DC, United States of America; University of Oklahoma Norman Campus: The University of Oklahoma, UNITED STATES

## Abstract

Mutualistic and commensal interactions can have significant positive impacts on animal fitness and survival. However, behavioural interactions between pelagic animals living in offshore oceanic environments are little studied. Parasites can negatively effect the fitness of their hosts by draining resources and diverting energy from growth, reproduction, and other bodily functions. Pelagic fishes are hosts to a diverse array of parasites, however their environment provides few options for removal. Here we provide records of scraping behaviour of several pelagic teleost species, a behaviour that is likely used for parasite removal. These records span three ocean basins and, to the best of our knowledge, include the first records of scraping interactions involving tunas, blue sharks, and mako sharks as well as the first records of intraspecific scraping. We found that scrapers preferred scraping their head, eyes, gill cover, and lateral surfaces, areas where parasites are commonly found and where damage would likely have a substantial impact on fitness. Scraper species varied in their scraping preferences with tunas scraping mostly on the posterior caudal margins of sharks and occasionally conspecifics, while rainbow runner scraped in more varied locations on both sharks and conspecifics. Lengths of scrapers and scrapees were positively correlated and fish scraping on sharks were larger than those scraping on conspecifics, suggesting that risk of predation may be a limiting factor. We show that pelagic teleosts prefer to scrape on sharks rather than conspecifics or other teleosts and suggest that this behaviour may have a positive impact on teleost fitness by reducing parasite loads. The decline of shark populations in the global ocean and the reduction in mean size of many species may limit these interactions, eroding possible fitness benefits associated with this behaviour, and consequently placing more pressure on already highly targeted and vulnerable species.

## Introduction

Symbiotic relationships and behavioural interactions among animals can provide a range of essential services and increase fitness and survival [1, 2]. These relationships range from obligate symbionts, where one species cannot survive without the other [3, 4], to shorter term behavioural interactions which still have a significant benefit to one or both parties [5–7]. If these relationships are eroded or the species involved are removed this can have a significant negative impact on the species of concern [8, 9]. Conversely, relationships between animals can have negative impacts on fitness, for example the detrimental impacts of parasites upon their hosts. Therefore, behavioural interactions which remove parasites can deliver benefits to the host [2]. The natural world is changing at an increasingly rapid pace, with widespread declines in wildlife [10, 11], climate change an ever growing presence [12], and human beings not only impacting terrestrial environments but the global ocean [13, 14]. As such the word’s wildlife and the connections among them are increasingly under threat.

Animals trade off energetic investment in growth, reproduction, and survival. Parasites directly and indirectly drain energy from the host and therefore diminish that available for other purposes, reducing the host’s fitness [15, 16]. These fitness costs can include loss of blood and nutrients, increased energy allocation to immune responses, risk of infection, impaired mobility and sensory performance, reduced competitive ability, and decreased food intake [17]. Even relatively light parasite loads can significantly reduce growth rates as well as body condition [18], and although a parasite load may not noticeably affect the host’s health, it may play a role in survival when there are intense demands on body resources, for example when escaping from predators or when under severe nutritional stress [19].

Parasites are found in marine environments where they often use fishes as hosts and can have similar impacts on their fitness to those documented for terrestrial animals [20]. Fish eyes, nares, inner ear, and lateral line are common sites of infestation which impact sensory performance [21]. Ectoparasites, those taxa that live on the external surface of hosts, can increase drag, leading to increased energetic expenditure, oxygen consumption, and weight loss [22]. Pelagic species in the open ocean can also have high parasite loads. Parasites associated with tunas have been studied relatively extensively due to the high level of exploitation, consumption, and farming of their hosts. These parasites utilise a wide range of niches in the body [23] and are associated with effects ranging from discomfort and tissue damage to mortality [24–26]. Farmed tuna also host a broad range of parasites [25], leading to husbandry efforts to reduce parasite loads [27].

As parasites can have a significant impact on the fitness of their hosts, natural selection has led to a range of adaptations to avoid, control, or eliminate them. These adaptations range from immune and other physiological responses to the use of specific structural and behavioural adaptations and symbiotic relationships that remove parasites directly [19]. Grooming is an important means of parasite removal in mammals and birds with dental combs, tongues, beaks, scratching with limbs, and picking with fingers all employed in the task [28, 29]. This raises the question of how fish, with no limbs, fingers, or other ability to scratch or pick at their body surface or otherwise reach the site of infestation can remove their parasites. Other methods must be employed with some viable options being employing another party, as primates do when allogrooming [28], or using suitable objects to scratch against as bears do with trees when they are unable to reach certain areas [30].

Cleaning stations allow fish to employ other organisms in the removal of their parasites. Cleaner wrasses and other symbiotic cleaner fishes and shrimps are known for their removal of parasites from fishes, sharks, turtles, and other marine animals visiting cleaning stations on reefs [31, 32]. Cleaner wrasses in particular can have a significant effect on the community ecology of reef fish populations, conferring a positive influence on host species size, abundance, and recruitment; therefore the severing of the relationship between host and cleaner has a negative impact on host fitness [2, 5]. However, these cleaning stations are not present in offshore waters and therefore mobile pelagic fishes must either make visits to these stations in coastal waters or look to alternative solutions. For example, sunfishes have been observed utilising albatrosses to remove their ectoparasites [33]. Fishes have also been observed scraping on substrates, anthropogenic structures, or other animals leading to suggestions that this behaviour is used to remove ectoparasites and dead skin [34]. Fishes in inshore environments have been observed scraping against sand [35], turtles [36], rays, and other fish [36, 37] with all studies suggesting parasite removal as the motivation. However, a large proportion of the records of scraping interactions described to date are of fishes scraping against sharks. Papastamatiou et al. [37] noted this behaviour in a reef environment, with rainbow runner *Elagatis bipinnulata* rubbing their flanks along the skin of grey reef sharks *Carcharhinus amblyrhynchos* at Kingman Reef, Line Islands and a single observation of a bluefin trevally *Caranx melampygus* repeatedly rubbing against a Galapagos shark *Carcharhinus galapagensis* at Midway Atoll in the Northwestern Hawaiian Islands. They concluded that the abrasive quality of the placoid scales of shark skin provides a surface, which when rubbed in a tail to head direction, would be effective in removing parasites, necrotic tissue, and other irritants. That these fishes are rubbing against a potential predator even in an environment where hard substrate is abundant suggests that the surface must be particularly suited to the task.

In offshore waters, options are much more limited, as are observations of scraping. Gooding and Magnusson [34] recorded pelagic fish such as the pompano dolphin fish *Coryphaena equiselis* chafing against their research raft as well as against an oceanic whitetip shark *Carcharhinus longimanus* in an apparent attempt to rid themselves of ectoparasites. These observations suggest that similar scraping behaviours are employed in the open ocean, however little is known about how other pelagic fishes might manage their parasite loads. The lack of available options in offshore waters would likely increase the value of any possible scraping substrate. Here we describe a variety of scraping interactions captured on baited remoted underwater video systems (BRUVS) that were recorded between pelagic fish and shark species. We investigate the qualitative and quantitative nature of these interactions and expand on existing theories explaining this behaviour. We also comment on possible impacts for the fitness of these species and conservation implications when the species involved in these interactions become scarce and the opportunity for these interactions to take place becomes limited.

## Materials and methods

### BRUVS dataset and video analysis

All data were extracted from a global dataset of 6,166 deployments of mid-water stereo-Baited Remote Underwater Video Systems (BRUVS), captured across 55 expeditions to 36 global locations between 2012 and 2019 (S1 Table). These data were collected as part of a broad programme investigating the status and ecology of pelagic wildlife communities. Footage recorded from these deployments was processed to estimate species richness, abundance, and fork length using standardised methods [38], returning records of 117,726 individuals from 261 taxa. Sample locations span the Pacific, Indian, and Atlantic oceans across 80 degrees of latitude and 333 degrees of longitude. Throughout this analysis, opportunistic behavioural observations were made. When scraping interactions were observed, they were noted so that they could be revisited for closer analysis.

On deployments where scraping was observed, the entire video was re-analysed to gather quantitative and descriptive data on the species and individuals involved. Each time an interaction was observed, we recorded: species, fork length, and individual, if possible, for both the animal scraping (scraper) and that being scraped (scrapee). Additional details of the interaction were recorded including which area of the scraper’s body was scraped and on which part of the scrapee this contact was made. A written description of the approach, contact, and departure was also made. We also recorded occasions where scrapers attempted to make contact with a scrapee and failed or abandoned the attempt. Where an individual could be tracked between multiple scrapes, a record was kept so that the nature of these interactions could be compared.

Fork length measurements were made of each individual involved in each interaction using stereo-photogrammetric calculations in the SeaGIS software EventMeasure (www.seagis.com). Individual measurements were taken when the animal was as close and perpendicular to the rig as possible to maximise the precision of measurements. Raw lengths were log_10_(x) transformed prior to analysis. Log(x) transformed lengths did not depart significantly from normal distributions (Shapiro-Wilk—shark-teleost scrapees p = 0.696, W = 0.951; shark-teleost scrapers p = 0.079, W = 0.869; conspecific scrapees p = 0.979, W = 0.980; conspecific scrapers p = 0.504, W = 0.944). We investigated the relationship of size of scrapers to scrapees using linear regression based on log_10_(x) transformed fork length of each unique scraper-scrapee pair for both shark-teleost interactions and interactions between conspecifics. Where multiple interactions occurred for a given scraper-scrapee pair, only the first interaction was included in length analyses to maintain independence. We compared the mean lengths of scrapers scraping on sharks and those scraping on conspecifics using a two-tailed t-test.

Based on the records of the number and success of scrapes, we tested whether scrapers were overall more likely to succeed in their attempt to scrape than not, using a chi square goodness of fit test with Yates’ correction for continuity [39]. The relative success of different scraper species was compared using a chi square contingency test on successful and failed attempts. Notes made on interactions were qualitatively compared within and between species to determine the variety or consistency of scraping method by characterising the approach, preparation, contact, and departure conducted by each species. The response of scrapees to contact or attempted contact from scrapers was also investigated for evidence of evasive action by searching the written descriptions of interactions. Evasive action was defined as having taken place in interactions where the scrapee was described as: altering direction away from contact, bending body to avoid contact, or accelerating away from the scraper.

The video analysis also allowed us to pinpoint the areas that were most targeted by the scraper and where on the scrapee’s body this contact occurred. We defined areas scraped as one or more of the following: left head/eye, right head/eye, left gill cover, right gill cover, ventral gill cover, left pectoral fin, right pectoral fin, left lateral surface, right lateral surface, ventral body surface, dorsal body surface, pelvic fins, dorsal fin, anal fin, and caudal fin (S1a Fig). For example, if a tuna contacted a shark on its left side, first with its gill cover and maintained contact over its pectoral fin and flank before contact ended, the record of surfaces scraped would include; left gill cover, left pectoral fin, and left lateral surface. We tested for overall preferences in scraping areas across all species using a chi-square goodness of fit and for differences among scraper species in the areas they scraped most often using a chi squared contingency test. Each scraper species was then tested to see whether all body surface groups were scraped at equal rates with chi-squared goodness of fit tests. To facilitate these analyses, we grouped body areas as: head/eye/gill-cover, lateral surfaces/pectoral fins, ventral surface/pelvic fins/anal fin, and dorsal surface/dorsal fin/caudal fin. Records of individuals which were observed to scrape multiple times were investigated further to determine whether scrapes targeted the same or different areas.

The part of the scrapee on which contact was made was also defined. Contact to the caudal fin was divided into categories based on which portion of the fin was contacted defined as: left or right, upper or lower lobe, and anterior or posterior margin. Other surfaces contacted were defined as: dorsal surface/dorsal fin, ventral surface, lateral body surface left, and lateral body surface right (S1b Fig). We tested for overall preferences in scraping location across all species with a chi squared test for goodness of fit, and for differences among scraper species in the preferred area on the scrapee they scraped using a chi squared contingency test. We also tested whether individual species showed significant preferences for scraping area on the scrapee using chi squared tests for goodness of fit.

### Global overlap of teleost scrapers and sharks

We investigated the degree of association between sharks and the species we observed scraping on them by looking at the overlap in their records from our global database. We determined the overlap of each scraper species observed with all shark species by comparing the number of deployments on which they were recorded together and the number where the scraper was recorded with no sharks present. We tested the association between sharks and scrapers by using chi squared goodness of fit tests to investigate whether scraper species overall were recorded significantly more often on deployments with sharks present than those where sharks were absent. A chi squared contingency test was used to determine whether this pattern of association differed by species; and if each species was more or less likely to be associated with sharks.

## Results

Eleven stereo BRUVS deployments were selected for analysis based on observations of scraping during preliminary analysis. Each of these deployments consisted of 2–3 hours of footage. These deployments were from three locations across three ocean basins: the Revillagigedo Archipelago in the tropical eastern Pacific (5 deployments); Ascension Island in the tropical central Atlantic (4 deployments); and Recherche Archipelago in the temperate Indian Ocean off south-western Australia (2 deployments; S1 Table).

**Fig 1:**
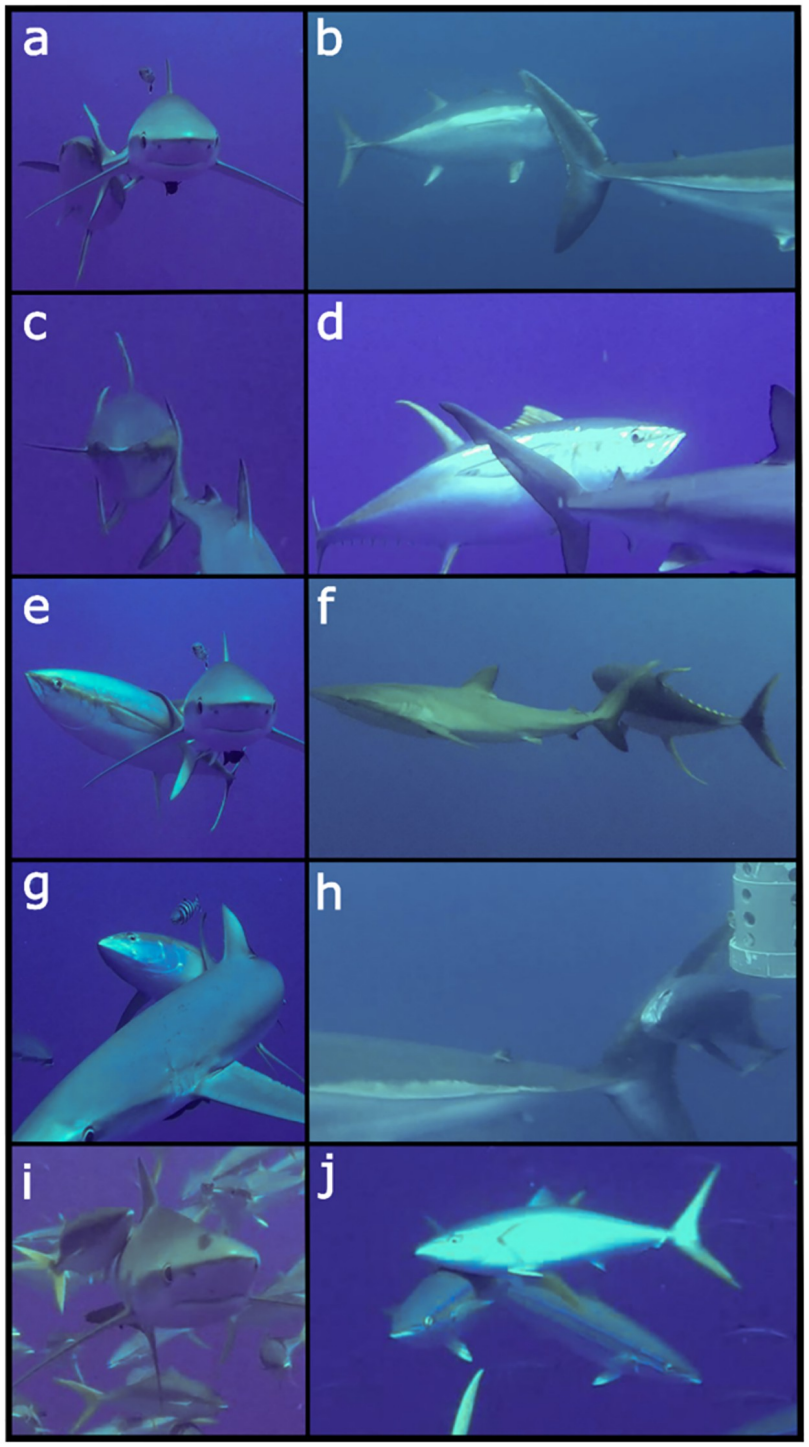
Example images of some of the scraping interactions observed. Anterior and lateral views of scrapees scraping their head (a, b), lateral surface/pectoral fin (c, d), dorsal surface (e, f), and ventral surface (g,h) on the posterior caudal margin of sharks. i) and j) show scrapers respectively scraping their ventral surface on the lateral surface of a shark and gill cover on the dorsal surface of a conspecific. Scraper-scrapee pairs are; yellowfin tuna *Thunnus albacares*—blue shark *Prionace glauca* at Ascension Island (a, c, d, e, g), southern bluefin tuna *Thunnus maccoyii*—shortfin mako shark *Isurus oxyrhinchus* at Recherche Archipelago (b, h), yellowfin tuna—silky shark *Carcharhinus falciformis*, Revillagigedo Archipelago (f), rainbow runner *Elagatis bipinnulata*—blue shark, Ascension Island (i), and rainbow runner-rainbow runner, Ascension Island (j).

Three species of tuna, one species of carangid, and three species of shark were involved in 106 interactions and scraping was observed both on sharks and conspecifics (Fig 1; Table 1). Species observed scraping were: three members of the family Scombridae, yellowfin tuna *Thunnus albacares* (44.3% of interactions), southern bluefin tuna *Thunnus maccoyi* (16%), and skipjack tuna *Katsuwonus pelamis* (3.8%); and one carangid, the rainbow runner *Elagatis bipinnulata* (35.8%). Shark species subject to scraping were: two requiem sharks (Family: Carcharhinidae), the blue shark *Prionace glauca* (58.5%) and silky shark *Carcharhinus falciformis* (11.3%); and one salmon shark (Family: Lamnidae), the shortfin mako *Isurus oxyrhinchus* (13.2%). Conspecifics were scraped in 17% of interactions. All scraper species utilised conspecifics and sharks with the exception of skipjack tuna which did not scrape on sharks. Blue sharks were scraped on by both rainbow runner (49–72.3 cm) and yellowfin tuna (47.8–150 cm) at Ascension Island and southern bluefin tuna (68.4–83 cm) at Recherche Archipelago. Silky sharks were scraped on by yellowfin tuna (78.1–202 cm) at both Ascension Island and Revillagigedo Archipelago. A single shortfin mako was scraped on by multiple southern bluefin tuna (59.4–78.8 cm) at Recherche Archipelago.

**Table 1:** Details of scraper and scrapee species recorded.

Common name	Species	Mean FL (range) scrapers (cm)	Records of scraping	% of records on sharks	Mean FL (range) scrapees (cm)	Records of being scraped	Common FL (cm)	Max FL (cm)	IUCN Status
yellowfin tuna	*Thunnus albacares*	131 (41.7–201.9)	47	96%	49.8 (43.4–56.2)*	2*	150	239	Near Threatened
southern bluefin tuna	*Thunnus maccoyii*	70.1 (59.4–83.0)	17	94%	73.8 (73.8)*	1*	160	245	Critically Endangered
skipjack tuna	*Katsuwonus pelamis*	40.3 (36.6–44.0)	4	0%	39.6 (37.4–42.0)*	4*	80	110	Least Concern
rainbow runner	*Elagatis bipinnulata*	54.6 (46.9–72.3)	38	71%	54.8 (51.7–58.5)*	11*	69.3	139	Least Concern
blue shark	*Prionace glauca*	-	-	-	174.7 (163.8–194.9)	62	275	329	Near Threatened
silky shark	*Carcharhinus falciformis*	-	-	-	169.4 (116.6–240.1)	12	192	269	Vulnerable
shortfin mako shark	*Isurus oxyrinchus*	-	-	-	175.8 (175.8)	14	250	413	Endangered

All lengths are fork lengths (FL) in cm. Asterisks indicate records of scrapes on conspecifics. Common FL and Max FL are reported from FishBase (Froese & Pauly 2019 [40]; accessed October 2020). IUCN Status is as reported on the International Union for the Conservation of Nature (IUCN) RedList of Threatened species website (www.iucnredlist.org; Accessed October 2020).

Across all global deployments of mid-water BRUVS, tunas were observed on 5% of deployments, rainbow runner were observed on 3%, and sharks were observed on 27%. Overall, deployments with scraper species in this study present, whether scraping behaviour was observed on that deployment or not, were significantly more likely to also have sharks present than not (67%; χ^2^_1,433_ = 51.3, p<0.001). Scraper species differed significantly in their level of association with sharks (χ^2^_3, 433_ = 141.7, p<0.001). Rainbow runner (83% with sharks; χ^2^_1,208_ = 91.6, p<0.001), yellowfin tuna (83%; χ^2^_1,96_ = 42.7, p<0.001), and skipjack tuna (71%; χ^2^_1,24_ = 4.17, p = 0.041) were all recorded significantly more on deployments with sharks than without, whilst southern bluefin tuna were observed significantly less with sharks than without (20%; χ^2^_1,105_ = 37.8, p<0.001). Of note was the close association of yellowfin tuna and silky sharks, with silky sharks present on 61% of deployments with yellowfin tuna recorded.

### Number and success of scrapes

**Fig 2:**
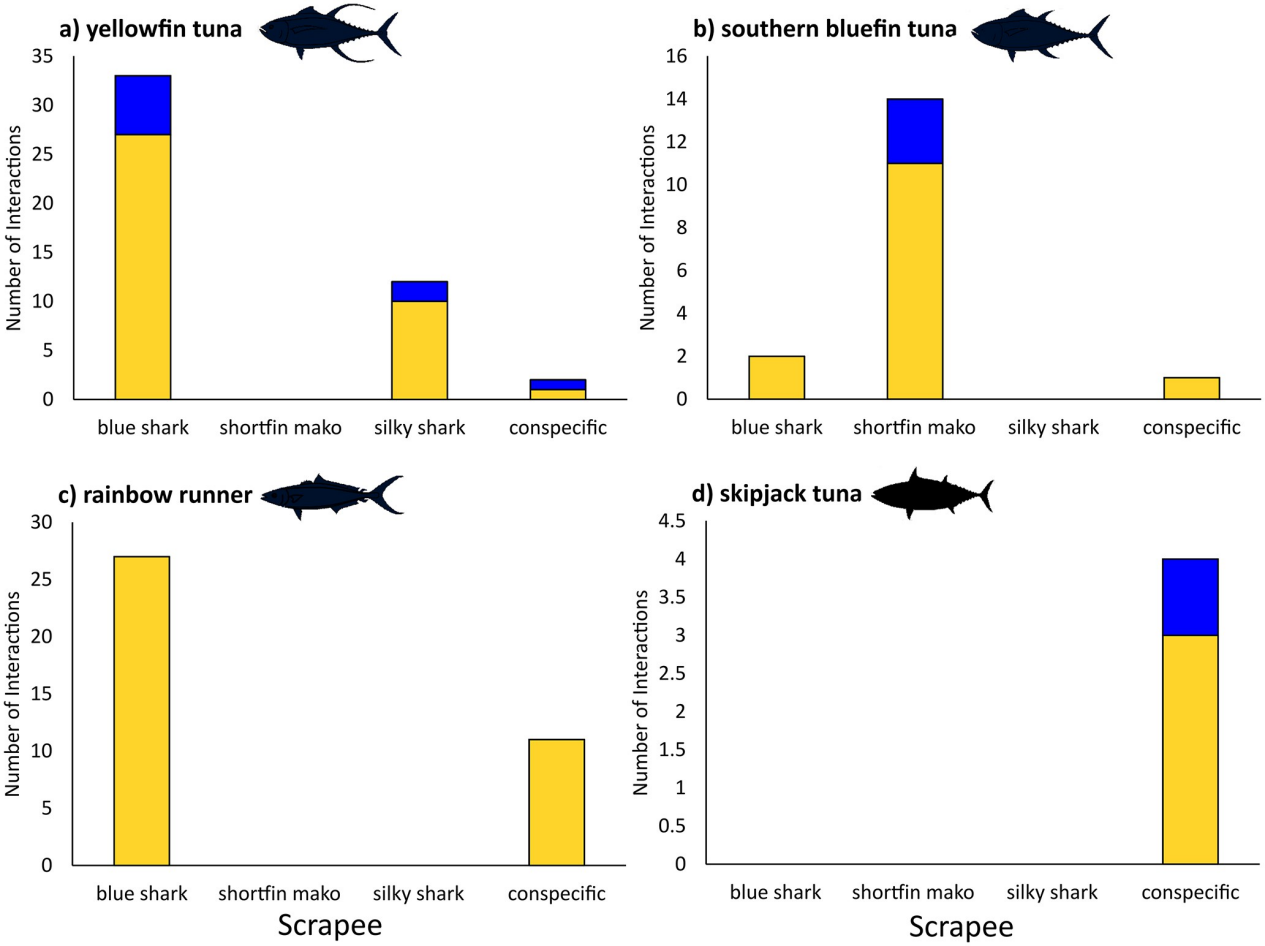
Number of successful (yellow) and unsuccessful (blue) scraping interactions by scrapers on scrapees across all sample sites for a) yellowfin tuna *Thunnus albacares*, b) southern bluefin tuna *Thunnus maccoyii*, c) rainbow runner *Elegatis bipinnulata*, and d) skipjack tuna *Katsuwonus pelamis*.

Overall, scrapers were significantly more likely to succeed in their attempt to scrape than to fail (χ^2^_1,106_ = 60.4, p<0.001) with 87.7% of interactions a successful scrape and the remaining 12.3% observations of following with missed or abandoned attempts of scraping. Scraper species differed significantly in the proportion of successful to unsuccessful interactions (χ^2^_2,102_ = 8.10, p = 0.019), with rainbow runner most likely to succeed (100%), and yellowfin and southern bluefin tuna abandoning or missing scrape attempts at higher rates (Fig 2). Of successful scrapes, 77 (83%) were on sharks with the remaining 16 (17%) on conspecifics. Of unsuccessful scrapes 11 (85%) were on sharks and 2 (15%) were on conspecifics, however these interactions differed qualitatively. For the unsuccessful interactions with sharks, in all cases the scraper followed the shark, began to position itself to scrape and typically abandoned the scrape attempt as the shark suddenly changed direction coming to the bait canister of the BRUVS. In contrast, the two unsuccessful attempts within conspecifics (yellowfin and skipjack tuna) involved noticeable evasive action taken by the scrapee-to-be, avoiding contact with the scraper. In addition to this, in scrapes between conspecifics where contact was made, noticeable evasive action was taken by the scrapee in 75% of cases, as opposed to zero observations of evasive action taken by sharks. Evasive action was taken in 100% of conspecific scrapes in tunas and 70% of observed scrapes within rainbow runner.

### Lengths

**Fig 3:**
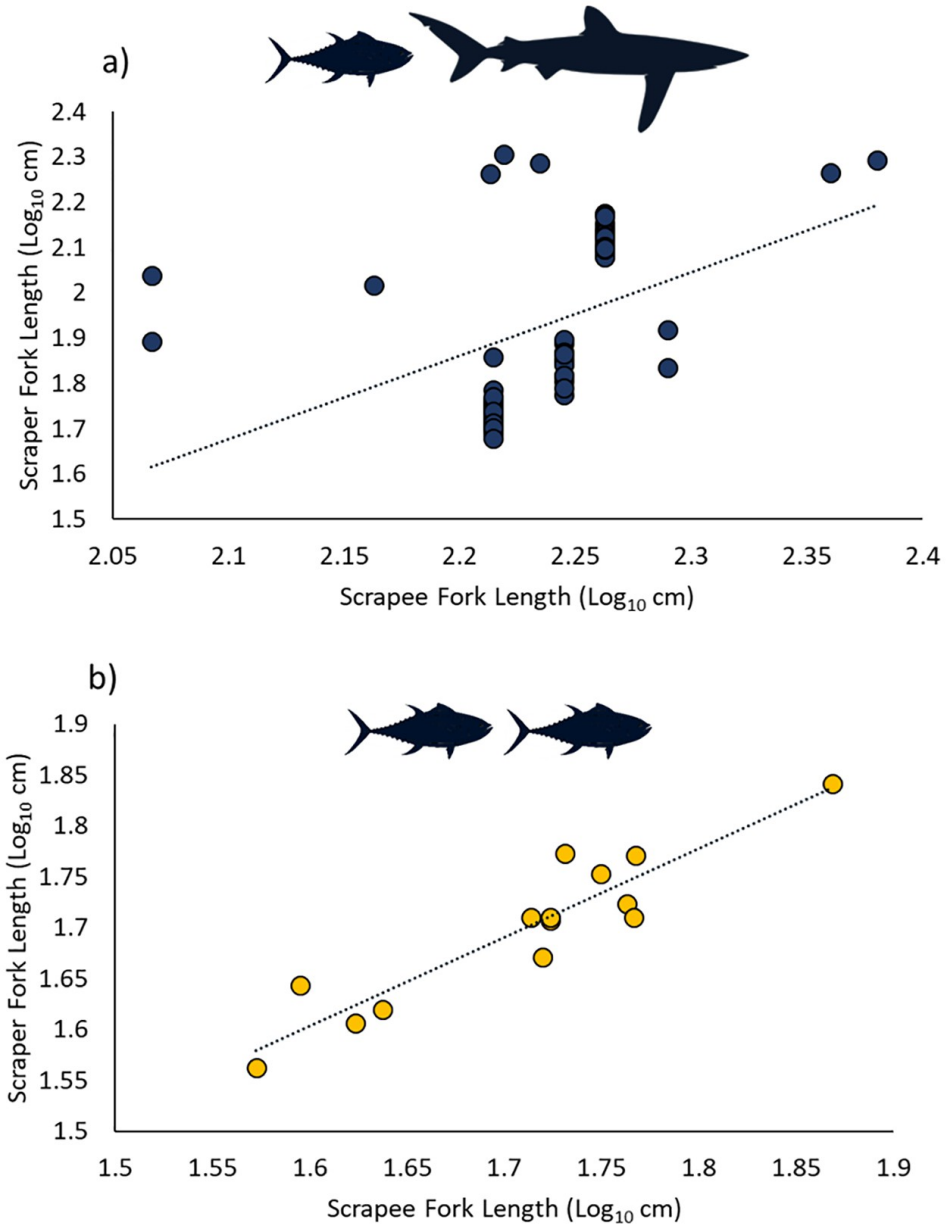
Relationship of scraper to scrapee fork length (log_10_(x) transformed) for a) teleost-shark (log_10_(Scraper FL) = 1.85 x log_10_(Scrapee FL)– 2.20; R^2^ = 0.185) and b) teleost-teleost conspecific (log_10_(Scraper FL) = 0.872 x log_10_(Scrapee FL)– 0.208; R^2^ = 0.861) pairs for independent observations of scraping interactions across all sample sites.

Scraping fish covered a broad range of lengths from a 36.5 cm skipjack tuna to a 202 cm yellowfin tuna. Scrapee sharks ranged in fork length from 117–240 cm. Lengths were available for 80 (75%) unique interactions. Teleosts scraping on sharks were on average significantly larger 94.0 cm ± 5.34 SE than those scraping on conspecifics 50.8 cm ± 2.32 SE (t_79_ = 3.66, p<0.001). The ratio of scraper to scrapee lengths for teleost-shark pairs ranged from 29% to 122% with a mean of 54% ± 2.8% SE. The smallest fish to scrape on a shark was a 47.8 cm southern bluefin tuna. There was a significant positive correlation between teleost-shark scraper-scrapee fork length (p<0.001, R^2^ = 0.185; Fig 3a). The relationship of scraper to scrapee fork length in conspecific interactions was tighter with scraper-scrapee length ratios ranging only from 88% to 112% with a mean of 98% ± 1.8% SE. There was a strongly significant positive correlation between fork length of conspecific scrapers and scrapees (p<0.001; R^2^ = 0.861; Fig 3b).

### Scraper areas

**Fig 4:**
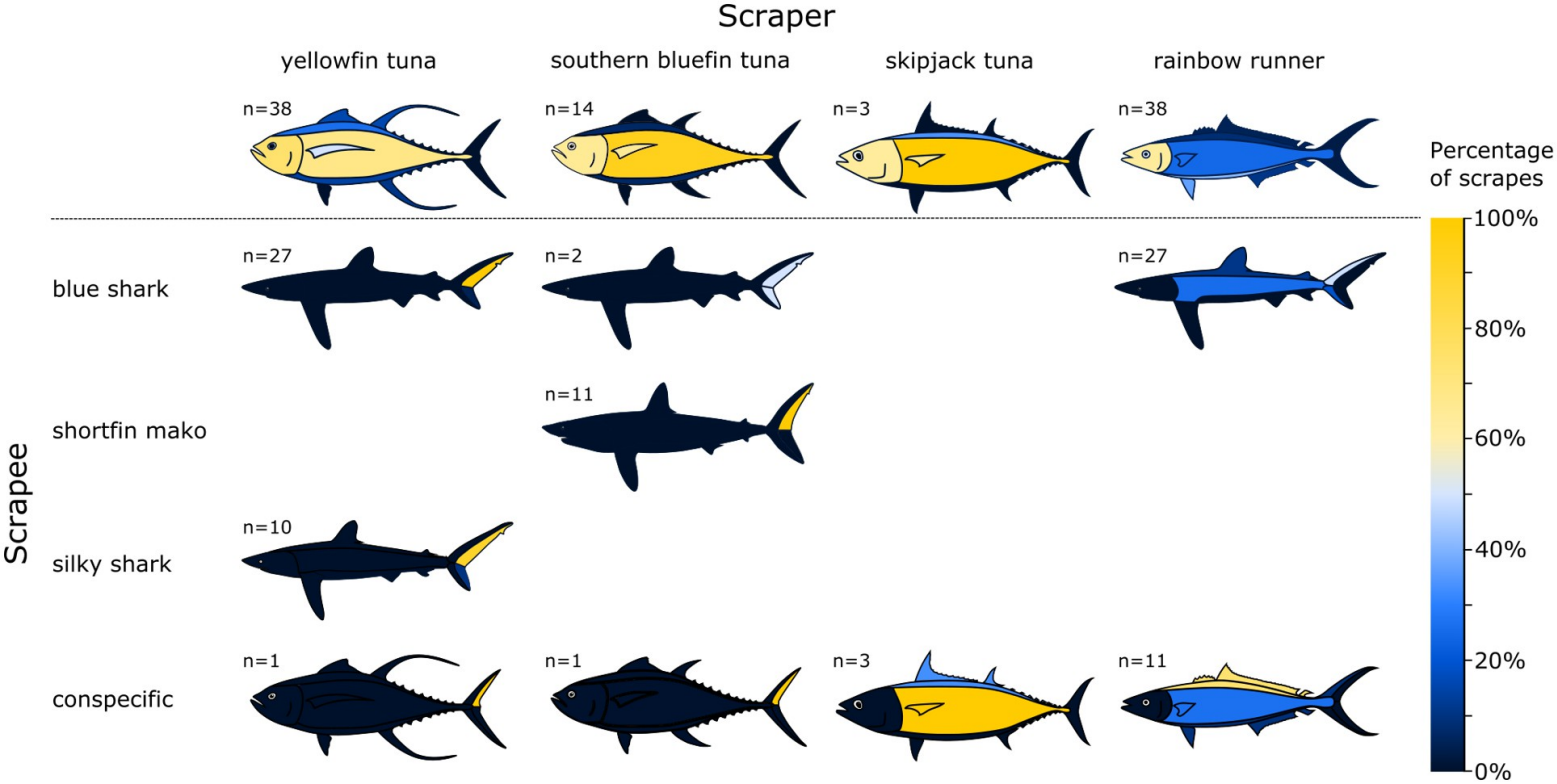
Most frequently scraped areas organised by scraper (vertical) and scrapee (horizontal) species. The upper panel shows the distribution of scraping areas on the scraper’s body with areas scraped at high proportions yellow and low proportions dark blue. The lower panel shows the areas on scrapees scraped by each scraper species again with areas contacted in higher proportions in yellow and lower proportions in blue. Number of interactions for each scraper-scrapee pair are indicated.

Out of 93 successful scrapes across all species, the head/eye/gill cover (68%) and lateral surfaces/pectoral fins (61%) were scraped most frequently with ventral surfaces/pelvic fins/anal fin scraped at a lower rate (27%) and dorsal surfaces/dorsal fin/caudal fin scraped at much lower rates (16%) (χ^2^_3,160_ = 41.7, p<0.001; Fig 4). Although there was a general pattern in scraping preferences across species, scraper species significantly differed in the areas they scraped most often (χ^2^_9,160_ = 16.9, p = 0.044). Three of four species scraped some areas of the body significantly more frequently than expected by this general pattern (Fig 4). Rainbow runner scraped their ventral surfaces/pelvic fins/anal fin and head/eye/gill cover more than expected, and their lateral body/pectoral fins less than expected (χ^2^_3,60_ = 12.4, p = 0.006). Southern bluefin tuna showed the opposite pattern, scraping their lateral surfaces/pectoral fins more than expected and the ventral surface/pelvic fins/anal fin and dorsal surface/dorsal fin/caudal fin less than expected (χ^2^_3,24_ = 18, p<0.001). Yellowfin tuna scraped the head/eye/gill cover area and lateral surfaces/pectoral fins more than expected and the ventral surface/pelvic fins/anal fin and dorsal surface/dorsal fin/caudal fin less than expected (χ^2^_3,70_ = 22.9, p<0.001). Only skipjack tunas, which had the smallest number of recorded interactions showed no variation (χ^2^_3,6_ = 3.33, p = 0.36).

Multiple scrapes by single individual scrapers were confirmed for 15 individuals: six yellowfin tuna, three southern bluefin tuna, and six rainbow runner. Of these multiple scrapes, 84% were on sharks and for one southern bluefin tuna these multiple scrapes were across both a shark and a conspecific. On 60% of occasions, repeat scrapers scraped more than one of their body surfaces (left, right, ventral, dorsal) with 5 of 6 yellowfin tuna scraping both their left and right sides and additionally scraping either their ventral or dorsal surface. Five individuals scraped the same area multiple times.

### Scrapee areas

Scrapers showed a strong overall preference for scraping the posterior margin of the caudal fin of the scrapee (χ^2^_2,98_ = 19.9, p<0.001). These preferences however, varied significantly among scraper species (χ^2^_6,145_ = 105, p<0.001; Fig 4). Yellowfin and southern bluefin tunas showed a significant (χ^2^_2,39_ = 78.8, p<0.001; and χ^2^_2,14_ = 28.3, p<0.001, respectively) and similar preference for where on the scrapee they scraped; they always scraped on the posterior margin of the caudal fin and almost invariably scraped on the upper caudal lobe of both sharks and conspecifics (98%, n = 51 obs). These tunas also showed a marked preference for scraping on sharks rather than conspecifics with 97% and 93% of scrapes on sharks, respectively. In contrast, rainbow runner showed a much more varied choice in where on the scrapee they scraped regardless of whether the scrapee was a shark or conspecific. They showed a significant preference for scraping on the anterior caudal fin and other body surfaces, over the posterior caudal (χ^2^_2,41_ = 21, p<0.001). On occasions when they scraped on the caudal fin (46%), they always passed in front of it scraping on the anterior edge or upper tip of the fin with 74% of caudal scrapes on the upper lobe and 26% on the lower lobe. Of scrapes on sharks, 64% were on the caudal, 25% on the lateral body surface, and 11% on the dorsal surface/fin. In contrast when scraping on conspecifics, only 8% (n = 1) of scrapes were on the caudal fin with the majority landing on the dorsal surface/fin 62% (n = 8) followed by the lateral 24% (n = 3), and ventral body surfaces 8% (n = 1). The preference for sharks, although present, was not as marked as in yellowfin and southern bluefin tunas with sharks scraped on 68% of occasions and the remaining 32% conspecifics. Skipjack tuna, scraping only on conspecifics, differed in their preferences, scraping significantly more on body surfaces and never on the caudal fin (χ^2^_2,4_ = 8.08, p = 0.019). With 100% (n = 3) of scrapes contacting the lateral surfaces and 25% (n = 1) also contacting the dorsal surface. In all these interactions the conspecific scrapee attempted to avoid contact with the scraper, likely altering the actual area contacted from the targeted area of contact.

### Qualitative descriptions of interactions

Scraper species differed qualitatively in their method of scraping in relation to their approach, preparation to scrape, contact, and departure. Yellowfin and southern bluefin tunas had a similar method of scraping on sharks and followed a strict set of steps leading to a scrape. These species always approached from the rear of the scrapee in line with the centre of the caudal fin. They then approached to within less than one body length of the shark, then stalled as the sweep of the shark’s tail approached an appropriate position. They then accelerated towards the shark with several fast tail beats. The area to be scraped was prepared by closing the gill cover and adducting any fins in the contact area. The tail of the shark was then contacted, usually just as it passed the midline, and the scraper arched its body to maintain contact over the scraping area as the shark’s tail swept across, wrapping around the body surface. They then turned sharply away from the shark and moved, usually at speed, away from it perpendicularly. The area of the scraper contacted could be adjusted by rotating and arching the body to contact the lateral, dorsal, or ventral surface (Fig 1c–1h). When multiple tuna were present, they would take turns to scrape forming a chain with the tuna that had just scraped usually returning to the end of the queue. Scrapes by rainbow runner on sharks were subjectively much less organised. The school generally formed around the posterior half of the shark and individual fish periodically moved out of the school and contacted the shark on various parts of its body (Fig 1i). The scrapers would then either move away or continue to make successive scrapes within a short period of time.

Scraping on conspecifics was more similar among species, most often consisting of an approach from the rear above, acceleration towards the scrapee, arching to maintain contact, and alteration of swimming direction by the scrapee. Conspecific scraping in yellowfin and southern bluefin tunas targeted the same areas as when scraping on sharks and consisted of the same steps although it usually started offset, above, below, or to one side of the rear of the scrapee and the scrapee always altered its swimming to make evasive action. Skipjack tuna scrapers always approached from the rear and above of their conspecifics, a position which seemed to be in the blind spot of the scrapee, they accelerated fast toward the scrapee which also accelerated, contact was made by rotating and curving away at the last moment to scrape down the lateral surface, noticeable displacement of the scrapee was evident on contact. Rainbow runner also tended to approach conspecifics from the rear above, accelerate towards them, and rotate or curve their body to control the contact area, scrapees almost always altered their swimming direction to avoid contact.

## Discussion

Our results show that several pelagic teleosts use scraping behaviour and that this behaviour may be used to remove parasites and other irritants. Animals, including mammals, rub their bodies in response to pain [41]. Fish demonstrably do the same, rubbing a site of induced pain against available substrates, with a lack of response if an analgesic is applied [42]. Scratching or rubbing a specific area against a suitable substrate is thus likely to be a response to pain or irritation and parasites are very capable of causing pain, irritation, and damage to fish. For example, copepod infestations in tunas can lead to ulceration, bleeding, lesions, and may even penetrate into the muscle [23]. We found that all tuna species tended to scrape their head and lateral body much more than the rest of their body and rainbow runner showed more varied scraping but still had a focus on the head, ventral, and lateral surfaces. These areas include many of the most common areas of ectoparasite infestation [43, 44] as well as the primary sensory organs, to which damage from parasites would have a substantial impact on fitness [17]. For example, the monogenean flatworm *Nasicola klawei* infests the nasal cavities of yellowfin tuna [45], the trematode flatworm *Platocystis viviparoides* is found in the lateral body especially along the lateral line in Atlantic bluefin tuna *Thunnus thynnus*, the copepods *Euryphorus brachypterus* and *Penella filosus* graze on the skin, gills, and muscle of several species, and the copepod *Brachiella thynni* is adapted to the cavity behind the pectoral fins [46]. Grazing by copepods over the tissue of the eye has been recorded, causing keratitis, panophthalmitis, and cataract formation [23]. This type of damage to the sensory performance of visual predators, such as tunas, would likely have a substantial negative impact on hunting success and therefore survival. Similarly, damage to the lateral line would impact sensory performance and damage to the gills would impact respiration. It is therefore highly likely that if scraping is a behaviour used to remove parasites, then these areas would be targeted as priorities for parasite removal.

Parasites of marine fishes are more abundant and their communities more diverse in warmer waters [47]. As well as parasite communities being more abundant in the tropics, large and typically older fish host more abundant and diverse parasite assemblages than small, younger fish [47, 48]. The largest scrapers in this study were yellowfin tunas, found at our tropical locations of Ascension and Revillagigedo, and although their primary areas of scraping were the same as the smaller bluefin and skipjack tunas, they also scraped areas that these species did not, notably the dorsal and anal fins. The increased range of areas scraped may reflect the higher abundance and diversity of parasites typical of these large fishes with a potentially increased range of niches inhabited on the body.

The fitness benefits of parasite removal are well established, and scraping may be a method of removal for pelagic fish. However, the question remains why sharks are a preferred subject on which to scrape given that they are predators of the scraper species. There are several possible theories as to why sharks may provide particularly suitable scraping surfaces. One suggestion, as Papastamatiou et al. [37] concluded, is that the abrasive quality of shark skin provides an ideal surface against which fish can remove parasites and dead skin. Shark skin is made up of small tooth like structures called dermal denticles; it feels like, and in pre-industrial times was used as, sandpaper [49]. This rough surface is therefore more suited to the task at hand than the relatively smooth skin of teleosts. Even within sharks, dermal denticle size and arrangement varies substantially and therefore some sharks are likely to have a more suited texture for scraping than others. Silky sharks are named for their relatively smooth skin and their very fine denticles may be the perfect emery board for fishes. It is also unlikely that sharks are used for scraping in the open ocean simply because they are the only option: we observed conspecific scraping at lower rates than shark scraping. Moreover, fish scrape on sharks in coastal reef environments where a variety of benthic surfaces and options such as cleaning stations exist to remove parasites [37]. These observations suggest that sharks are a preferred option against which to scrape.

Sharks may make a preferable scraping surface due to their relatively large, long, and flexible caudal fins, and their relatively slow and predictable tail beat. The limited number of conspecific scrapes observed in this study, as well as the nature of abandoned scrape attempts as sharks changed direction, suggest that specific attributes may be required in a scrapee to make a successful scrape. The constant, predictable, and relatively slow swimming motion of sharks may facilitate scrapers to time the sweep of the tail to maximise contact and the length and flexibility of the tail may help to sustain contact around the curve of the body surface (Fig 1c and 1e). The lack of reaction to scraping is also likely a factor, as no sharks observed in this study showed any visible reaction to scraping and did not move to avoid contact or respond aggressively, whereas conspecifics consistently made efforts to avoid contact.

Conspecific animals are scraped at a lower rate than sharks despite their higher availability. This suggests that they are a less-ideal surface for scraping. There are a number of factors that likely contribute to their lower suitability. Evasive action would make scraping more difficult for a would-be scraper and may also alter the contact area of the scrape likely reducing its effectiveness. Teleost skin is less rough than that of sharks and covered with a layer of mucous making it slippery [50] which would reduce its effectiveness in dislodging parasites and dead skin. This smooth skin may explain the pattern in scraping areas on conspecifics, with rainbow runner targeting the dorsal surface most, and yellowfin and southern bluefin tunas scraping the caudal fin. These areas have hard fin structure against which a parasite may be removed. Skin mucous may also help to explain the evasive behaviour observed as this layer provides a buffer to outside stresses and has protective antimicrobial and antiparasitic properties [51]. Scraping may act to remove this protective layer on the scrapee and lead to increased chances of infection. Another possible reason why evasive action was observed in conspecifics and not in sharks is to avoid possible direct transfer of parasites. If ectoparasites were removed from the scraper they may be able to re-attach to the scrapee. Obligate ectoparasites tend to be highly host specific [52], explaining why sharks do not evade contact. For mobile, fast moving pelagic fishes like tunas the probability of a minute parasitic larvae finding its host seems low, however genetic studies in tuna parasites have suggested that multiple collisions between parasite larvae and tunas best explain the genetic structure present [45]. Schooling behaviour has been suggested as a possible mechanism for the facilitation of this horizontal dispersal, however, direct contact between conspecifics, such as we observed, would likely increase the chances of transfer and, if so, avoidance of contact would be in the best interests of a conspecific scrapee’s fitness.

The tight association between sharks and tunas [53, 54] likely increases their utility as a scraping subject. We found close associations between three of the four species in this study and sharks. Yellowfin tuna, skipjack tuna, and rainbow runner were more likely to be recorded with sharks than without. There was also a particularly tight relationship between yellowfin tuna and silky sharks. This association would, over time, provide the opportunity for behavioural relationships, such as those we describe, to develop. Although bluefin tuna showed some overlap with sharks, this association was not as tight as that of other species. This could be due to the colder waters they inhabit, with lower parasite levels and therefore a reduced reliance on sharks as a mechanism to remove parasites. A contributing factor may also be the threatened status of both scraper and scrapee species. Southern bluefin tuna are listed on the International Union for the Conservation of Nature (IUCN) Redlist of Threatened Species as Critically Endangered [55] with their shark scrapees listed as Endangered [shortfin mako; 56] and Near Threatened [blue shark; 57]. The probability of bluefin tuna and these scrapees encountering one another and interacting is likely diminished by their reduced abundance.

All scraper species except skipjack tuna scraped on sharks significantly more than conspecifics. This raises the question of why skipjack tuna did not scrape on sharks even though they were present and are generally a preferred subject on which to scrape compared to conspecifics. A likely reason is the risk of predation. We showed that scrapers scraping on sharks were significantly larger than those scraping on conspecifics. Studies based on stomach content analyses and gape width of sharks have indicated that 75% of shark prey is less than 20% of shark length [58] and no more than 36% of their own body length [59, 60]. Skipjack tuna lengths were 22.6–25.7% of the length of sharks recorded on their samples which falls within this suggested prey size range. The mean scraper-scrapee length ratio for teleost-shark pairs in our records was 54% ± 2.85 SE with a minimum of 29% (Fig 3a). Shortfin mako and blue sharks are known to eat tunas [61], and silky sharks are specifically recorded eating yellowfin tuna, skipjack tuna, and rainbow runner [62]. There is thus likely a cut-off length ratio below which the trade-off between parasite removal and predation risk leads teleosts to not scrape on sharks. Conspecifics and smaller sharks may be more important scraping options for these smaller fishes. As skipjack tuna commonly reach 80 cm and a maximum of 110 cm, it may be that they utilise sharks for scraping at these larger sizes. This risk of predation may also help explain why scrape attempts are sometimes abandoned after the initial set-up, as a certain set of conditions may be needed for these fish to complete a successful scrape without unacceptable predation risk.

Our records expand the range of species involved in scraping interactions and indicate that these interactions are more common than reported. First, there are few direct observational methods that are applied to pelagic habitats and their wildlife, with mid-water BRUVS being regularly deployed only in the last 5 years [38]. This lack of sampling means that there are very few direct behavioural observations of pelagic animals in their natural environment, however, the recent application of BRUVS to offshore waters has led to a rapid accumulation of high quality footage facilitating the study of pelagic animal behaviour [63, 64]. Second, these interactions may be seldom reported as large predators are relatively scarce and therefore less likely to be observed together. Shark populations have declined by over 90% in some regions [65–67], and populations of tunas and their relatives have been reduced by 60% on average over the past half century [68, 69]. Further records will help to determine the range of species involved in these interactions and therefore the extent of animals affected if these behavioural connections are lost. The use of remote video systems has been expanded from standard metrics to include a range of new outputs from the footage collected [63, 70–72]. BRUVS are a useful tool for behavioural observations as they allow large amounts of footage to be collected, increasing the chances of recording rare events and allow these events to be replayed and analysed in detail through post-processing. BRUVS also allow sampling in offshore waters where diving is not practical. Future BRUVS work should include records of potentially ecologically important behaviours such as those we present here.

We have shown that the association between pelagic fishes and sharks is an important one, likely for the removal of parasites, which if left unchecked, can have significant negative impacts on fitness. The continued decline of global shark populations may thus have a knock-on effect on pelagic fish populations. Pelagic fishes live in a patchy environment with many predators and where food supply can be volatile; climate change is increasing pressure on energetic requirements by reducing oxygen availability [73, 74]. Tunas have very high metabolic rates compared to other teleosts [75] and therefore have very large energy maintenance demands [76]. The effect of parasites in combination with these factors may act to drastically reduce fitness. The fact that we only observed these behaviours in remote areas away from large human populations where these highly commercially targeted predators are relatively abundant serves to illustrate that these behavioural links may be eroded through the removal of both or either party. This inference is supported by the scraping behaviour records of Papastamatiou et al. [37] and Gooding and Magnuson [33] which were all made in very remote areas. In the latter case, the records were made from a research raft in the offshore waters of the Pacific in the 1960s before global commercial fisheries catches began to decline [69]. Reductions in populations of teleosts and sharks may also reduce other associative benefits, such as those brought about by group foraging [77, 78]. The erosion of these relationships may have a negative impact, not only on the health and survival of individual fishes but may have economic implications for humans. Higher parasite loads may decrease the body condition of these fishes reducing their quality as a food resource. Behavioural associations among marine species are complex and often little studied however they may have important conservation implications. Human impacts reduce fish populations and disrupt their behaviour. However remote regions and marine protected areas, where human pressures are lightest, have been shown to preserve the behaviour of fishes and sharks [79, 80]. Therefore, implementation of large MPAs and improved fisheries management may help to preserve important behavioural relationships, such as those we describe, and the possible associated fitness benefits.

## Supporting information

S1 Fig Schematic of a) defined body areas scraped on the scraper’s body and b) defined areas for contact by scrapers on the scrapee’s body. (TIF)

S1 Table Locations sampled with mid-water BRUVS by the Marine Futures Lab from 2012–2019. Number of mid-water BRUVS deployments at each sample locations and number of deployments on which scraping behaviour was observed on preliminary analysis.
(DOCX)

S1 File Records of interactions. (XLSX)
