# Supplementary figures and images for: Sharks are the preferred scraping surface for large pelagic fishes: Possible implications for parasite removal and fitness in a changing ocean
Source: PLoS One. 2022 Oct 19;17(10):e0275458. doi: 10.1371/journal.pone.0275458 (PMC9581428; doi:10.1371/journal.pone.0275458)

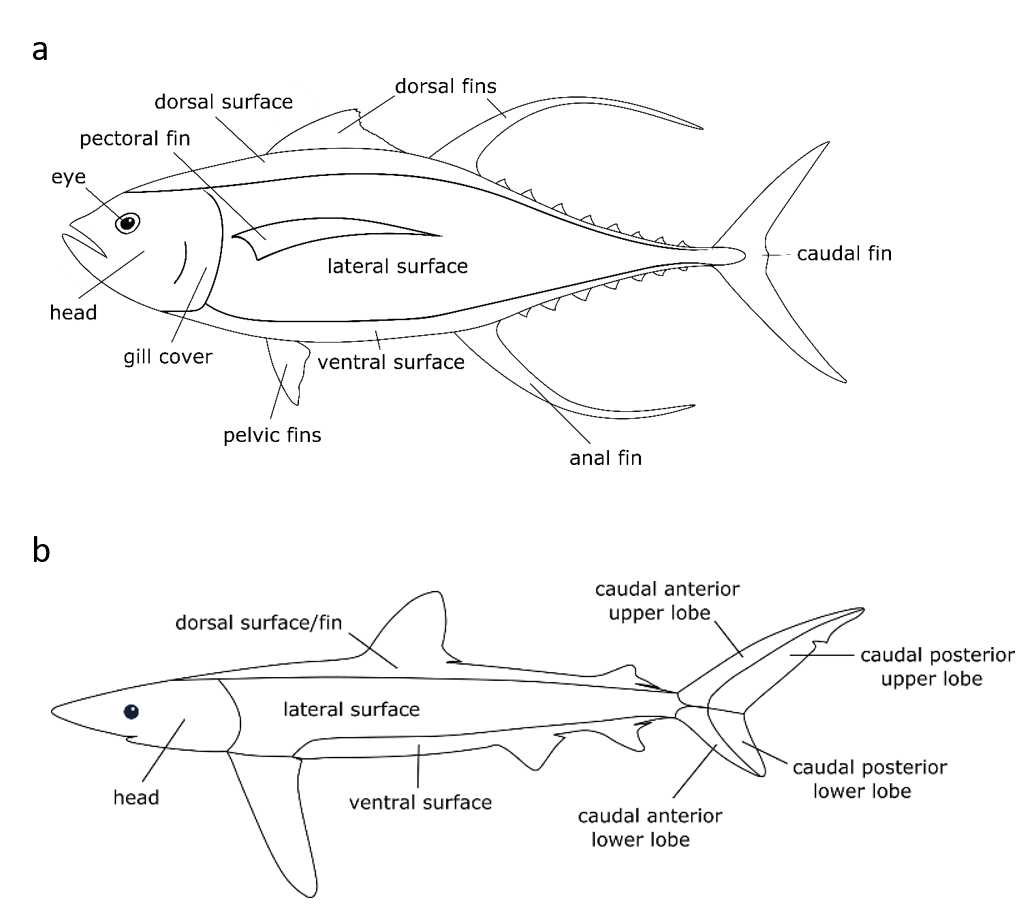

Supplement: S1 Fig — (TIF) [file pone.0275458.s001.tif]
