# Supplementary material for: Sharks are the preferred scraping surface for large pelagic fishes: Possible implications for parasite removal and fitness in a changing ocean
Source: PLoS One. 2022 Oct 19;17(10):e0275458. doi: 10.1371/journal.pone.0275458 (PMC9581428; doi:10.1371/journal.pone.0275458)
Supplement: S1 Table — Number of mid-water BRUVS deployments at each sample locations and number of deployments on which scraping behaviour was observed on preliminary analysis. (DOCX) [file pone.0275458.s002.docx]

**Supplementary Table 1**: Locations sampled with mid-water BRUVS by the Marine Futures Lab from 2012 – 2019. Number of mid-water BRUVS deployments at each sample locations and number of deployments on which scraping behaviour was observed on preliminary analysis.

| **Location** | **Country** | **Latitude** | **Longitude** | **Deployments (scraping obs)** |
| --- | --- | --- | --- | --- |
| Argo Terrace | Australia | -15.4 | 118.5 | 240 |
| Ascension Island | United Kingdom | -8.4 | -13.9 | 655 (4) |
| Azores | Portugal | 39.1 | -30.2 | 155 |
| Bremer Canyon | Australia | -34.7 | 119.7 | 200 |
| Chagos Archipelago | United Kingdom | -6.1 | 72.2 | 546 |
| Clipperton Island | France | 10.3 | -109.2 | 51 |
| Cocos Island | Australia | -12.1 | 96.8 | 110 |
| Far North Queensland | Australia | -11.3 | 143.4 | 164 |
| French Polynesia | French Polynesia | -20.9 | -137.9 | 50 |
| Geographe Bay | Australia | -33.5 | 115.2 | 350 |
| Gracetown | Australia | -34.0 | 114.8 | 300 |
| Maldives | Maldives | 6.0 | 72.9 | 205 |
| Malpelo Island | Colombia | 4.0 | -81.6 | 85 |
| Montebello Islands | Australia | -20.1 | 115.4 | 200 |
| New Caledonia | France | -20.2 | 164.5 | 160 |
| Ningaloo Reef | Australia | -21.8 | 113.8 | 280 |
| Niue | Niue | -19.5 | -168.8 | 100 |
| North West - Ashmore Reef | Australia | -12.2 | 123.1 | 200 |
| North West- Long Reef | Australia | -13.9 | 125.7 | 200 |
| Osa Peninsula | Costa Rica | 8.6 | -83.8 | 169 |
| Palau | Palau | 7.4 | 134.5 | 147 |
| Perth Canyon | Australia | -32.0 | 115.1 | 419 |
| Pilbara | Australia | -20.1 | 116.3 | 535 |
| Rapa Iti and Marotiri | French Polynesia | -27.7 | -144.1 | 53 |
| Recherche Archipelago | Australia | -34.1 | 123.2 | 330 (2) |
| Revillagigedo Archipelago | Mexico | 19.0 | -111.1 | 75 (4) |
| Rowley Shoals | Australia | -17.1 | 119.4 | 55 |
| Ilhas Selvagen | Portugal | 30.1 | -15.9 | 57 |
| Shark Bay | Australia | -26.2 | 113.1 | 342 |
| Timor | Australia | -11.8 | 127.2 | 120 |
| Tonga | Tonga | -22.0 | -157.1 | 36 |
| Tristan da Cunha | United Kingdom | -37.2 | -12.4 | 63 |
| Tristan da Cunha - Gough Island | United Kingdom | -40.3 | -9.9 | 18 |
